# Cisplatin-Induced Skeletal Muscle Dysfunction: Mechanisms and Counteracting Therapeutic Strategies

**DOI:** 10.3390/ijms21041242

**Published:** 2020-02-13

**Authors:** Elena Conte, Elena Bresciani, Laura Rizzi, Ornella Cappellari, Annamaria De Luca, Antonio Torsello, Antonella Liantonio

**Affiliations:** 1 Department of Pharmacy-Drug Sciences, University of Bari, 70125 Bari, Italy; elena.conte@uniba.it (E.C.); ornella.cappellari@uniba.it (O.C.); annamaria.deluca@uniba.it (A.D.L.); 2 School of Medicine and Surgery, University of Milano-Bicocca, 20900 Monza, Italy; elena.bresciani@unimib.it (E.B.); laura.rizzi@unimib.it (L.R.); antonio.torsello@unimib.it (A.T.)

**Keywords:** cisplatin, skeletal muscle, ghrelin, growth hormone secretagogues (GHS), muscle atrophy

## Abstract

Among the severe side effects induced by cisplatin chemotherapy, muscle wasting is the most relevant one. This effect is a major cause for a clinical decline of cancer patients, since it is a negative predictor of treatment outcome and associated to increased mortality. However, despite its toxicity even at low doses, cisplatin remains the first-line therapy for several types of solid tumors. Thus, effective pharmacological treatments counteracting or minimizing cisplatin-induced muscle wasting are urgently needed. The dissection of the molecular pathways responsible for cisplatin-induced muscle dysfunction gives the possibility to identify novel promising therapeutic targets. In this context, the use of animal model of cisplatin-induced cachexia is very useful. Here, we report an update of the most relevant researches on the mechanisms underlying cisplatin-induced muscle wasting and on the most promising potential therapeutic options to preserve muscle mass and function.

## 1. Introduction

Cis-diamine-dichloroplatinum (II) (best known as cisplatin or CDDP) is a very effective cancer drug having a major clinical impact, particularly for patients with bladder, head and neck, lung, ovarian, and testicular cancers. Cisplatin is intracellularly activated by the aquation of one of the two chloride leaving groups, and thereafter it binds covalently to DNA adducts. DNA modifications activate various intracellular pathways including those involved in DNA-damage recognition and repair, cell-cycle arrest, and programmed cell death/apoptosis [1,2]. Cisplatin-based chemotherapy is associated with severe side effects, including nephrotoxicity, ototoxicity, neurotoxicity, and muscle wasting. In particular, a significant body weight loss is often present in cancer patients treated with cisplatin, mainly due to muscle wasting. Despite its toxicity even at low doses, cisplatin remains the first-line therapy for several types of solid tumors. Therefore, the necessity of developing more effective and safe chemotherapeutic adjuvants or nutritional supplements to ameliorate cisplatin-induced muscle wasting is crucial. Preserving muscle mass could have a high impact on the patients’ quality of life. The definition of molecular pathways underlying cisplatin-induced muscle dysfunction is fundamental for identifying targets with high therapeutic potentials. In this setting, the use of animal models to evaluate in vivo cisplatin effects is very valuable. Cancer and chemotherapy may cause the appearance of a cachectic phenotype by activating the same or similar mechanisms. Accordingly, cisplatin administration to healthy rats can reproduce some of the alterations typical of cancer cachexia, including body weight loss, adipose tissue remodeling, skeletal muscle wasting, and weakness. Furthermore, it is also well known that molecular mechanisms controlling muscle wasting in chemotherapy and cancer share common features with pathophysiology of other relevant muscle neurodegenerative such as muscular dystrophies [3,4]. The animal model commonly used to study the derangements specifically associated with chemotherapy and for testing new therapies for cachexia is the rat or mouse intraperitoneally treated with cisplatin (1–3 mg/kg) for 3–4 consecutive days to induce weight loss without over-toxicity [5,6,7,8,9]. Body weight and food intake of these animals are significantly reduced following cisplatin administration. In particular, the final body weights of cisplatin-treated animals show a reduction of about 30% [5,6,7,8,9]. These adverse effects appear already from the first day of cisplatin administration and remain evident for the duration of the experimental protocol [8,9]. After cisplatin administration is halted, cisplatin-treated animals restart gaining weight and increase their food intake, but their body weight remains significantly lower than that of control animals [5,8,9].

Herein, we report an overview of the molecular mechanisms underlying muscle wasting induced by cisplatin-damaging chemotherapy, also focusing on the potential therapeutic strategies that have been suggested to counteract cisplatin-related toxicity at skeletal muscle level.

## 2. Molecular Mechanism Underlying Cisplatin-Induced Muscle Wasting

The balance between protein synthesis and degradation ensures the physiological muscle proteins turnover. The most common symptoms associated with cisplatin administration in patients with cancer are muscle weakness and fatigue mainly due to skeletal muscle mass depletion [10].

**Figure 1:**
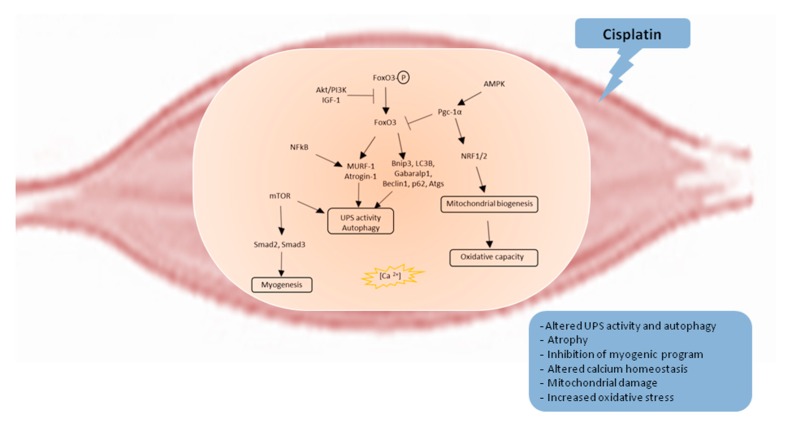
Molecular mechanism underlying cisplatin-induced muscle wasting. AMPK: 5’-adenosine monophosphate-activated protein kinase; Akt: Protein kinase B; Atgs: Autophagy-specific genes; Bnip3: BCL2 interacting protein 3; FoxO3: Forkhead boxO, Gabaralp1: GABA type A receptor-associated protein-like 1; IGF-1: Insulin-like growth factor-1; IL-1: Interleukin-1; IL-6: Interleukin-6; LC3B: Lipidated microtubule-associated protein 1 light chain 3 alpha; mTOR: Mammalian target of rapamycin; MuRF-1: Muscle ring-finger-1; NFκB: Prototypical nuclear factor kappa light-chain-enhancer of activated B cells; NRF1/2: Nuclear respiratory factor 1/2; p62: Sequestosome-1; PGC-1α: Peroxisome proliferator-activated receptor γ coactivator-1α; PI3K: Phosphoinositide 3-kinases; Smad2/3: Small mother against decapentaplegic.

Cisplatin-induced muscle dysfunction is caused by the activation of several mechanisms ranging from the alteration of ubiquitin-proteasome, autophagy and insulin-like growth factor-1 (IGF-1)/PI3K/Akt pathway to calcium homeostasis and lipid metabolism dysregulation, mitochondrial damage, oxidative stress and pro-inflammatory cytokines upregulation (Figure 1).

### 2.1. Ubiquitin-Proteasome Pathway

Weight loss characterizing cachexia involves a reduction of protein synthesis and the activation of several proteolytic systems, such as those calcium-dependent and ubiquitin-dependent, and the lysosomes [11]. Ubiquitin-dependent proteolysis is the main mechanism for the increase degradation of muscle protein in cancer cachexia [12]. Ubiquitin-proteasome pathway (UPP) is characterized by reactions involving three classes of proteins: Ubiquitin-activating enzymes (E1), ubiquitin-conjugating enzymes (E2), and ubiquitin protein ligases (E3) [13]. Muscle atrophy F-Box (MAFbx)/atrogin-1 and muscle ring-finger-1 (MuRF-1), are two muscle-specific E3 ubiquitin ligases that are increased in skeletal muscle under atrophy-inducing conditions and considered markers for muscle atrophy [14,15]. Cisplatin-treated animals develop significant muscle atrophy and weakness followed by an increased expression of the ubiquitin ligases MAFbx/atrogin-1 and MuRF-1 systems [7,8,9]. This activation is partially due to dephosphorylation of the transcriptional factor Forkhead boxO (FoxO3a) [16], which is in turn regulated by the pivotal mediator Akt and regulates genes coding for the autophagy pathway contributing to the degradation of muscle proteins, finally promoting atrophy [17]. MyoD and myogenin, two markers of muscle differentiation, also play an important role in counteracting cancer-induced cachexia by stimulating muscle regeneration. However, their activities are dependent on Akt phosphorylation, p38, myostatin (Mstn), and TNF-α levels [17,18]. Cisplatin administration induces a significant reduction of MyoD and myogenin expression [19]. Muscle mass is regulated through an interplay between anabolic and catabolic pathways. The IGF-1/PI3K/Akt/mammalian target of rapamycin (mTOR) pathway along with the UPP and Mstn pathway maintain this homeostasis supported by various transcriptional factors [20]. The activation of Mstn signaling, a member of the transforming growth factor beta (TGF-β) family, synthesized and secreted predominantly from skeletal muscle fibers, causes muscle atrophy [21]. The upregulation of Mstn has been recently associated with muscle wasting in cancer cachexia and after cisplatin administration. Particularly, Mstn inhibits Akt signaling through the activin type II receptor, and therefore the downstream mammalian target of rapamycin (mTOR) pathways that lead to protein synthesis, resulting in the activation of a Smad2 and Smad3 transcription factor complex, which mediates myogenesis genes inhibition, leading to skeletal muscle atrophy [22,23,24]. It has been also proposed that the catabolic muscle protein breakdown observed in cachexia could be due primarily to an increase in the expression and activation of the prototypical nuclear factor kappa light-chain-enhancer of activated B cells (NF-κB) [25]. In skeletal muscle, when a signal of tissue injury is present, toll-like receptors are activated, leading to an inflammatory response which terminates in NF-kB nuclear translocation. Moreover, TNF-α and other pro-inflammatory cytokines, which are major mediators of atrophy in skeletal muscle are produced [26]. Indeed, NF-κB may lead to muscle wasting by upregulating the expression of various proteins and inflammatory mediators involved in UPP, as well as impairing the myogenic program associated with the regeneration of atrophied skeletal muscle fibers [27]. Chemotherapy, including cisplatin treatment, induces NF-κB activity which is associated with muscle wasting in the absence of MuRF-1 induction [28,29]. It has been demonstrated that cisplatin-induced DNA damage causes the activation of NF-κB through the phosphorylation of its p65 subunit [29].

### 2.2. Autophagy Pathway

Autophagy is fundamental to maintain intracellular protein homeostasis in skeletal muscle [30]. Among the several components underlying the autophagy multistep pathway, the levels of lipidated microtubule-associated protein 1 light chain 3 alpha (LC3) form (LC3-II) and of p62 are widely used as indexes to evaluate the activation of autophagy [31]. Furthermore, the FoxO3a factor has a pivotal role for autophagy activation in skeletal muscle [32]. As it is well known, the localization of the FoxO3a factor can be in the cytoplasm or into the nucleus, depending on its phosphorylation state mediated by Akt [33]. Active Akt phosphorylates FoxO3a on residues Thr32 and Ser253 leading to its inhibition by cytosolic retention via various binding proteins [34]. When Akt is inactive instead, FoxO3a remains in its dephosphorylated state and can migrate to the nucleus to activate the transcription of autophagy- or atrophy-related genes such as Murf1, Bnip3, and several autophagy-specific gene proteins (Atgs), including LC3B, Gabarapl1, and Beclin1, thus promoting autophagosome formation and muscle wasting [33,35]. In animal models of cisplatin-induced cachexia, it has been shown that cisplatin treatment in fast-twitch skeletal muscle stimulated cell autophagy by decreasing the level of active Akt and therefore, phosphorylated FoxO3a, thus favoring its localization in the nucleus. Accordingly, a parallel upregulation of genes coding for autophagy proteins such as Beclin-1, a protein involved in autophagy starting, and LC3-II and p62 has also been observed [36]. A similar effect has been also associated to cisplatin in an in vitro study performed on C2C12 myotubes [37].

### 2.3. Calcium Homeostasis

Calcium ions (Ca^2+^) are an intracellular messenger necessary for physiological cellular processes. In skeletal muscle fibers, Ca^2+^ signaling plays a pivotal role in the regulation of the excitation–contraction coupling (ECC) process, as well as in the regulation of the activities of several Ca^2+^-regulated enzymes and transcription factors, finally impacting on muscle mass composition and mitochondrial dynamics [38]. The two main players in skeletal muscle ECC process are the ryanodine receptor (RYR1), the sarcoplasmic reticulum Ca^2+^ release channel, and the voltage-sensitive calcium channel (DHPR). RYR1 is located on the sarcoplasmic reticulum (SR) junctional face membrane, and DHPR is located on the plasmalemma. Transverse tubules (T-tubules), which are plasmalemmal invaginations, run deep into the muscle fiber. The junctional SR membrane contains RYR1, as well as many other smaller proteins which ensure the maintenance of the structural integrity of the Ca^2+^ release apparatus. The binding of Ca^2+^ released from the SR to troponin C allows actin and myosin interaction, causing muscle contraction. The activation of sarcoplasmic/endoplasmic reticulum Ca^2+^ATPase (SERCA), together with RYR1 closure, ensures the termination of the contraction cycle and the consequential muscle relaxation. It has been widely demonstrated that store operated calcium entry (SOCE), a phenomenon mainly sustained by the stromal interaction molecule (Stim1) and the Ca^2+^ release-activated Ca^2+^ modulator 1 (Orai1), is also essential to ensure proper intracellular Ca^2+^ handling for muscle function [39,40]. Indeed, upon depletion of the Ca^2+^ store in the SR, cells take up extracellular Ca^2+^ to replenish the cytosolic stores via SOCE. Maintenance of muscle cell Ca^2+^ homeostasis is crucial for sustained contractility, as evidenced by a clear link between Ca^2+^ homeostasis alterations and muscle performance decline observed in several pathophysiological conditions affecting skeletal muscle. Accordingly, the patho-mechanism underling cisplatin-induced cachectic effect also involves a dysregulation in Ca^2+^ handling. In particular, resting [Ca^2+^]_i_ of EDL muscle after cisplatin administration was 2-fold increase compared to the one registered in control rats [9]. This effect was associated with a reduced response to the application of a depolarizing solution or caffeine, and to a reduced SOCE. Expression genes analysis also revealed that cisplatin reduced expression pattern of genes related to Ca^2+^ homeostasis apparatus, such as *Dhpr*, *Ryr1*, *Orai1,* and *Stim1* [9]. Other than interfering with ion channels function regulating cell excitability [9,41,42], calcium homeostasis alteration has generally an important impact on enzymes activity. Intracellular Ca^2+^ overload can be considered a cause of the increased protein degradation and muscle atrophy characterizing cisplatin-induced cachexia. Indeed, increased intracellular Ca^2+^ activates calpains, calcium-activated proteases, that play a pivotal role in the initiation of most proteolytic pathways such as the ubiquitin-proteasome pathway [43]. Furthermore, several studies show that intracellular calcium increase stimulates the mitochondrial apoptotic process through the activation of proapoptotic protein Bax which favors the formation of the mitochondrial permeability transition pore (mPTP) allowing the release of cytochrome C from mitochondria and promoting cell apoptosis [44].

Finally, all these effects induced by cisplatin could contribute to Ca^2+^ overloading in the cytoplasm of muscle cells, detrimentally interfering with muscle maintenance and function. Indeed, the disruption of Ca^2+^ homeostasis could impair the functionality of Ca^2+^-dependent proteases and phospholipases that are essential for various muscle functions [45,46]. Thus, calcium dysfunction could be strictly related to cisplatin-induced muscle impairment.

### 2.4. Mitochondrial Biogenesis and Dynamic

The peroxisome proliferator-activated receptor γ coactivator-1α (PGC-1α), the nuclear respiratory factor 1 (NRF-1), and the mitochondrial transcription factor A (TFAM) represent the three main proteins involved in mtDNA replication and maintenance [47,48]. PGC-1α is a nuclear coactivator acting as a master regulator of mitochondrial biogenesis and activating, among others, the expression of NRF-1. NRF-1 is a nuclear transcription factor that operates the expression of many mitochondrial genes, including TFAM. Particularly, TFAM is a mitochondrial protein controlling mtDNA maintenance and replication, as well as mitochondrial transcription [47,48]. Cisplatin treatment induced a decline of mitochondrial biogenesis and mitochondrial mass in rat tibialis anterioris (TA) muscle [36]. Indeed, levels of PGC-1α, NRF-1, and TFAM decreased in rats treated with cisplatin to an extent ranging from 30% to 50% compared to control animals. The change of PGC-1α might be due to the impairment of the PI3K-Akt-mTOR signaling pathway induced by cisplatin in this cachectic animal model [36]. Accordingly, changes in mtDNA levels were also observed [36]. Mitochondria are highly dynamic organelles that continuously change their morphology by fission and fusion. These two events are coordinated and necessary to satisfy the variable needs of cells [49]. Dysregulated mitochondrial dynamics have been reported in various diseases including cancer, and can cause oxidative stress, inflammation, and cell death [50]. Mitofusins 1 and 2 (MFN1 and 2) are crucial molecules for mediating fusion of mitochondrial outer membranes and tethering the outer membrane to the endoplasmic reticulum (ER). The main regulator of mitochondrial fission process is the dynamin-related protein 1 (Drp1), which translocates from cytosol to mitochondrial outer membrane upon activation. Phosphorylation of Drp1 can dictate its activation status. In particular, the phosphorylation at Ser-637 inhibits Drp1 activity thus preventing mitochondrial fission, whereas phosphorylation at Ser-616 activates Drp1 activity and induces mitochondrial fission [51]. Skeletal muscle fibers of cisplatin-treated animals are characterized by an increase of both fusion and fission proteins, with a prevalence of fission compared to fusion [36]. Indeed, a decrease in the level of Drp1 phosphorylated at S637 site has been observed, indicating an enhancement of fission activity of the protein leading to mitochondrial fragmentation, which is typically associated with atrophic muscles characterizing various metabolic disorders.

### 2.5. Oxidative Stress and Pro-Inflammatory Cytokines

Reactive oxygen species (ROS) production increases in various pathophysiological conditions such as in muscle atrophy [52]. It is well known that in the tumor microenvironment increased ROS levels coexist with hypoxia. Mitochondria are the main source of physiological and pathological cellular ROS production. During the oxidative phosphorylation process, mitochondria use oxygen to generate ATP from organic fuel molecules, but they also generate ROS. Two proteins are involved in ROS metabolism: (1) Peroxiredoxin III (PRX III); and (2) mitochondrial manganese superoxide dismutase (MnSOD). Peroxiredoxins (PRXs) are amongst the most abundant cellular antioxidants displaying high reactivity toward H_2_O_2_, a key mediator of redox signaling [53]. Under high levels of H_2_O_2_, hyperoxidation of PRX to its sulfinylated (PRX-SO2) and sulfonylated (PRX-SO3) forms leads to enzymatic inactivation and inhibition of peroxidase function favoring the increase of intracellular ROS levels [53]. The superoxide dismutase (SOD) family is important in oxidative stress modulation and MnSOD is the primary antioxidant that scavenges superoxide generated within the mitochondria [54]. During cancer chemotherapy, oxidative stress-induced lipid peroxidation generates numerous electrophilic aldehydes that can attack many cellular targets. Cisplatin chemotherapy has been associated with increased production of mitochondrial muscle ROS, demonstrated by the presence of an increased level of PRX-SO3 proteins and a decreased level of mitochondrial PRX III and MnSOD [36]. Oxidative stress can increase inflammation by stimulating the production of inflammatory cytokines including tumor necrosis factor-α (TNF-α), interleukin-1 (IL-1), interleukin-6 (IL-6), and interferon-γ (IFN-γ). Physiologically, the role of pro-inflammatory cytokines is necessary to balance anabolism and catabolism and to maintain the correct myogenesis process. However, in cancer or in muscle wasting, increased pro-inflammatory expression leads to activation of p38 and downregulation of Akt [55], thus triggering a destructive metabolism in skeletal muscles. Cisplatin induces TNF-α and IL-1 overexpression causing IkB kinase (IKK) complex activation, and leading to the phosphorylation of the NF-κB-bound inhibitors of NF-κB (IκBs), which are consequently ubiquitinated and degraded. This leads to nuclear translocation of activated NF-κB which then induces the expression of MAFbx/atrogin-1 and MuRF1 favoring muscle wasting and cachexia [56]. At the same time, IL-6, whose expression is also induced by cisplatin, can bind its receptor IL-6R causing homodimerization of gp130. This homodimerization activates the associated JAKs (Janus kinases) resulting in the activation of transcription factors of the signal transducer and activator of transcription (STAT) family suppressing protein synthesis [55,56].

### 2.6. Lipid Metabolism

Lipid homeostasis plays a critical role in energy metabolism and body weight control. Many studies have shown that activation of lipolysis [57,58] and reduction of de novo lipogenesis [59,60] contribute to adipose cachexia and weight-loss in cancer [58], whereas the inhibition of lipolysis protects against cancer-associated weight loss and muscle wasting [61]. The alteration of fat metabolism is often caused by cancer, but is further aggravated by chemotherapeutic agents such as cisplatin that induced fat atrophy, increased lipolysis and lipid oxidation, and a decrease in lipogenesis. Other than in white adipose tissue (WAT) and liver, these effects occur also in skeletal muscles. Indeed, in all these peripheral tissues cisplatin suppresses both fatty acid synthase (FAS), a key enzyme in de novo lipogenesis, and stearoyl coenzyme A desaturase-1 (SCD-1), the enzyme that catalyzes the rate-limiting step in the biosynthesis of polyunsaturated fatty acids (FA). At the same time, in WAT cisplatin increases carnitine palmitoyl transferase-1(CPT-1), the key regulatory enzyme for fatty acids [62]. The adipocyte fatty acid binding protein (also known as aP2) plays an important role in macrophage activation during inflammation [63,64]. Moreover, it is also able to enhance lipolysis and fatty acids mobilization by interacting with the hormone sensitive lipase (HSL) and decreases de novo lipogenesis [65]. In WAT, cisplatin induces an overexpression of aP2 and HSL stimulating lipolysis [62]. Finally, cisplatin induces an increase in liver and WAT β-oxidation, raising the level of CPT-1α, a marker of β-oxidation. Based on the critical role played by WAT and liver in energy homeostasis, cisplatin-induced WAT and liver dysfunction also influence muscle function [65]. Energy metabolism in muscle is pivotal to maintain a normal physiologic function given that contractility is highly dependent on muscle ability to synthesize and use lipids as a source of energy.

## 3. Therapeutic Strategies for Counteracting or Minimizing Cisplatin-Induced Muscle Wasting

The loss of skeletal muscle is a key adverse event induced by cisplatin therapy, often accompanied by a reduction in appetite, increased catabolism, and lowering in body weight. These effects are a major cause for a clinical decline of patients, since it is a negative predictor of treatment outcome and it is directly associated with increased mortality [66]. Effective pharmacological treatments for this condition are urgently needed.

### 3.1. Ghrelin

Ghrelin, an octanoylated 28-amino acid peptide secreted mainly by the stomach, is the endogenous ligand of the growth hormone secretagogues receptor 1 a (GHS-R1a). Its administration increases not only GH secretion but also food intake and body weight in animals and humans [67,68,69]. Ghrelin effectively prevents muscle atrophy induced by dexamethasone and angiotensin II [70,71]. The observation that also unacylated ghrelin, a ghrelin derivative which does not bind to GHS-R1a, exerts a protective effect on muscular functions, strongly supports the idea that ghrelin effects are also independent from GHS-R1a activation [70].

Based on its orexigenic and neuroprotective properties, ghrelin and other agonists of the GHS-R1a have been proposed as potential therapies for cancer cachexia. They could improve anorexia, muscle mass and strength, and weight loss in patients with cancer, particularly those receiving cisplatin-based chemotherapy [62,72]. Importantly, by using two different animal models of cachexia, Garcia et al. convincingly demonstrated that ghrelin prevents tumor- and cisplatin-induced muscle wasting through multiple mechanisms of action [19]. As described above, the cachectic condition is characterized by the activation of p38/C/EBP-β, Mstn, and inflammatory cytokines, and a decrease in Akt and myogenin/MyoD ultimately leading to increased proteolysis, decreased muscle mass, and strength. Ghrelin prevents muscle atrophy by downregulating inflammation, p38/C/EBP-β/Mstn, and by increasing Akt phosphorylation and activating myogenin and MyoD. These changes appear, at least in part, to target muscle cells directly. Ghrelin administration in this setting is associated with improved muscle strength and survival [5,19].

As previously reported, lipid metabolism plays a critical role in energy homeostasis and body weight regulation. Ghrelin prevents cisplatin-induced weight and fat loss by modulating de novo lipogenesis in liver, WAT, and muscle [62].

### 3.2. Growth Hormone Secretagogues

The acronym GHS (growth hormone secretagogues) indicates a large family of synthetic compounds with a heterogeneous chemical structure, which includes peptidyl, peptidomimetic, and nonpeptidic moieties. GHS, named also as growth hormone releasing peptides (GHRP), were developed in the late 1970s for their capability to stimulate GH secretion, both in vitro and in vivo [73,74,75]. Originally, the great interest towards their endocrine activity ensued from the lack of knowledge of the natural hypothalamic hormone that promotes GH secretion, the endogenous GH-releasing hormone (GHRH), discovered only in the 1982 [76]. About twenty years later it was found out that GHS mimic the activity of ghrelin [77,78,79], which exerts its biological actions by binding to GHS-R1a [80]. The GHS-R1a is a G-coupled receptor, member of the Gq/i family, activating the phospholipase C (PLC) and inducing intracellular Ca^2+^ mobilization [80]. As well as ghrelin, GHS activities are mainly mediated by acting on the GHS-R1a, although different research groups strongly agreed with the assumption of the existence of more than one receptor [81,82,83,84]. GHS share with ghrelin many endocrine and extra-endocrine activities, targeting both peripheral tissues and central nervous system (CNS). Novel GHS have improved pharmacokinetics profiles, major stability, better bioavailability, and higher CNS penetrance, characteristics that make them better candidates than ghrelin for drug development. Reportedly, GHS enhance GH secretion [81], exert anti-inflammatory [85,86,87] and anti-convulsivant actions [88], increase food intake, body weight, and lean body mass (LBM) [81,89], play a role in the regulation of bone metabolism [90], display effects on gastric acid secretion and gastric emptying [91,92,93], and possess protective activity on the cardiovascular system both in vitro [94,95] and in vivo, both in animals [96,97,98] and humans [99,100]. GHS also prevent skeletal muscle damage in muscle wasting conditions associated to cancer cachexia [101,102,103] and/or cancer chemotherapy [8,9,36]. Indeed, because of positive effects on energy balance, ghrelin or GHS are considered a possible treatment option for all these cachexia-related conditions. Studies performed with ghrelin are summarized in Section 3.1; here, we provide a viewing of the investigations focused on GHS. Among the GHS’s family, two molecules have been investigated to evaluate their potential beneficial effect on cisplatin-induced muscle damage: Hexarelin and JMV2894 [8,9,36]. Hexarelin, an old GHS [104], and JMV2894, a novel peptidomimetic derivative [89], are widely investigated for their pleiotropic actions, including the acute stimulation of GH secretion and food intake. In cisplatin-treated rats both the GHS, but in particular JMV2894, are able to inhibit the cisplatin-impact, improving body weight gain without significantly affecting food intake or the adipose tissue deposition, but rather increasing muscle mass therefore avoiding its atrophy [8,9]. Regarding the morphology of muscle tissue, the GHS increased the cross-sectional area of myofibers, which is an important measure of skeletal muscle plasticity. Moreover, it is also able to reduce the size of the damaged area, usually expressed as the sum of necrotic area, the amount of inflammatory cells infiltration and nonmuscle area. The latter is characterized by the deposition of wasting material and debris, which replace muscle tissue during the atrophic process [9]. The muscle protective effects displayed by hexarelin and JMV 2894 are corroborated also by gene expression analysis that reveals the downregulation of MuRF1 mRNA levels [8,9], a key component of proteasome system that plays important roles in muscle catabolism [105], and an upregulation of PGC-1α, a marker of muscle oxidative phenotype [106]. Ex vivo experiments made on extensor digitorum longus (EDL) muscles of cisplatin-treated rats suggested that GHS could boost skeletal muscles functionality, measured in terms of electrophysiological and Ca^2+^ handling machine properties [9]. Although to a different extent, both GHS and JMV 2894 effectively prevented all cisplatin-induced calcium homeostasis alterations, such as changes in resting cytosolic Ca^2+^, voltage-dependent and caffeine-induced calcium release, and SOCE [9]. GHS also modified the expression profile of genes related to calcium homeostasis machinery. Interestingly, all the beneficial activities mediated by hexarelin and JMV2894 have a significant impact on muscle function as proven by in vivo functionality measurements showing an increase of forelimb force in cisplatin + GHS-treated rats in comparison with cisplatin only treated rats [9]. This outcome is very important since the clinical studies with anamorelin in nonsmall cell lung carcinoma patients did not show any increase of handgrip strength [107]. GHS could exert protective actions on skeletal muscle also by modifying mitochondrial biogenesis and dynamics. Indeed, GHS administration can prevent mitochondrial deficiency in cisplatin-induced cachectic muscles by stimulating mitochondrial biogenesis and cellular antioxidant defenses, by ensuring the maintenance of the mitochondrial fission and fusion balance and by decreasing the accumulation of oxidized proteins [36]. The GHS beneficial impact could be mediated by direct and indirect effects on skeletal muscles. It should be specified that the molecular mechanisms activated by hexarelin and JMV2894 at skeletal muscle level have not been fully characterized. Thus, it is likely that, similarly to ghrelin, they may activate multiple pathways, and their effects could occur also in the absence of the GHS-R-1a receptor. All the positive effects mediated by GHS in the cisplatin-induced model of cachexia strongly support the idea that GHS could represent a therapeutic option to preserve muscle function in cachectic patients. HM01, a novel orally available GHS-R1a agonist, is endowed of a high affinity for the GHS-R1a, good brain permeability, and a longer plasma half-life compared with ghrelin [93]. HN01 has also been tested in different tumor-bearing hosts [101,102,103] characterized by reduced muscle protein synthesis due to catabolism activation, as a result of a complex inflammatory, endocrine, and nutrition-related effect [108]. In rat Morris-7777 hepatoma tumor model, chronic subcutaneous HM01 injections antagonized tumor anorexia and body weight loss, reduced muscle wasting, by enhancing gastrocnemius, soleus, and total muscle mass [101]. Similarly, in mice bearing colon-26 (C26) tumors, chronic HM01 oral administration significantly increased body weight and muscle mass [102]. In this case, the effects on muscle mass were not correlated with an activation of the E3 ligase pathway, because HM01 did not affect MuRF-1/MAFbx gene expression [102]. Furthermore, Z-505, another highly selective GHS-R1a agonist, significantly attenuated muscle wasting in tumor-bearing C-26 mice and inhibited the progression of cachexia symptoms [103]. The anabolic effects of Z-505 on muscle are likely not direct, since the GHS-R1a mRNA has not been detected in skeletal muscle, but are rather mediated by the increased plasma levels of IGF-1 and insulin [103] that in turn, could affect the E3 ubiquitin ligases system, as previously demonstrated in vitro in C2C12 myotube cells [109].

### 3.3. D-Methionine

D-methionine (D-met), the dextro-isomer of the methionine, is a sulfur-containing micronutrient, essential for life, as it can serve as a precursor of cysteine, taurine, and glutathione (GSH) via trans-sulfuration [110]. D-met is contained in egg albumin [111]. The first researches on D-met properties focused on its efficacy in preventing cisplatin-induced ototoxicity in rat by its specific ability to counteract oxidative stress, without affecting the tumor response to an antineoplastic agent [112,113,114,115,116]. The D-met antioxidant activity is displayed also in other areas of the body: (i) in the liver, preventing the decrease of mitochondrial glutathione levels [117]; (ii) in the CNS, improving neurogenesis of hippocampal neurons [118]; (iii) in the kidney, through elevation of antioxidative activities [119]; and (iv) in the gastrointestinal system, protecting the mucosa and the gut microbiome from cisplatin-induced imbalance [120]. Recently, some evidences in the rat showed that D-met could alleviate skeletal muscle wasting caused by cisplatin, opening new perspectives in taking care of common and heavy side effects associated with anticancer platinum-based chemotherapy [121]. Pretreatment with D-met results in a significant increase of muscle mass, coupled with an improvement of muscle atrophy as demonstrated by a better morphometric phenotype, characterized by increased myofiber diameter and cross-sectional area. The myoprotective action is partially due to a limitation in FOXO-1 and atrogin-1 mRNA overexpression [121]. The in vivo outcome of D-met administration is confirmed by in vitro experiments. Indeed, in C2C12 cells cisplatin reduces myogenin and MyoD mRNA levels, an effect that is inhibited by 24-h incubation with D-met [121]. These first experimental evidences support a D-met protective action against muscle atrophy/muscle mass loss; however, the research on D-met modulation of muscle atrophy is still at embryonic stage and further studies are mandatory to strengthen this hypothesis.

### 3.4. Taurine

Taurine (2-aminoethanesulfonic acid) is a natural amino acid present as a free form in many mammalian tissues where it is an essential regulator of intracellular osmolarity. Taurine endogenous synthesis is variable between individuals and depends on nutritional state, amount of protein intake, and cysteine availability. The intracellular concentration of taurine ranges between 5 and 20 μmol/g wet weight in many tissues, especially in excitable ones, such as brain, heart, and skeletal muscle. The concentration of taurine is 100-fold lower in plasma compared to tissues, suggesting that it is indeed required as a modulating key for cellular functions. The primary source of taurine in humans is diet and the estimated intake is 40–400 mg/day [122]. Taurine is commonly known for its effects as energizer and anti-fatigue compounds and it is present in many energy soft drinks including supplement cocktails for athletes. Taurine supplementation modulates autophagy and reduces both ER stress and apoptosis induced by cisplatin in renal cell [123].

Skeletal muscle is a tissue capable of concentrating the largest amount of body’s taurine, through the presence of a specific active transporter that uptakes taurine inside cells against concentration gradients [124]. In skeletal muscle, taurine stabilizes phospholipids in the sarcolemma, regulates Ca^2+^ and Cl^−^ channel activity, and limits exercise-induced weakness [124,125,126]. In addition, through yet unclear mechanisms taurine may control muscle metabolism and gene expression. Importantly, a study performed on C2C12 cells demonstrates that taurine pretreatment rescues myotubes from cisplatin-induced atrophy and regulates the activity of the autophagy-lysosome system by maintaining proper perinuclear autophagic vesicles and mitochondria size and density [127]. This promising taurine action in vitro in preventing cisplatin-induced muscle atrophy opens the way to in vivo molecular and biochemical studies aimed to define taurine impact on muscle homeostasis.

### 3.5. ACVR2B/Fc

Skeletal muscle size is negatively regulated by a set of secreted growth factors belonging to the transforming growth factor (TGF)-β superfamily, including GDF8, also known as Mstn, growth differentiation factor-11 (GDF11), and activins [128,129]. The activins (A, B, and AB) are proteins closely related to inhibin, presenting effects functionally opposite to those induced by inhibin. Importantly, other than inducing follicle-stimulating hormone (FSH) biosynthesis and secretion [130,131], activins show several biological activities regarding embryonic, as well as differentiated cells [132]. In particular, activin A has been shown to affect myotube differentiation [133] and negatively regulate muscle mass [19]. All these ligands initiate their effects by binding to activin receptor type 2B (ACVR2B), a serine–threonine kinase receptor, which dimerizes with Alk4/5 and signals intracellularly via Smad2/3 [132]. Genetic deletion of Mstn, ACVR2B, and Smad3 in mice leads to a significant increase of skeletal muscle mass [19,128,133,134,135]. Thus, for treating conditions characterized by loss of muscle mass and strength in humans, considerable efforts have been made to develop therapeutics to antagonize ACVR2B signaling [136,137,138,139,140]. ACVR2B/Fc is a soluble ACVR2B fusion protein and activin 2B receptor signaling inhibitor. Other than potently counteracting muscle wasting induced by FOLFIRI and doxorubicine, ACVR2B/Fc was effective in counteracting muscle toxicity induced by cisplatin treatment [141].

Table 1 summarizes the promising potential therapeutic options and the related mechanism of action.

## 4. Conclusions

Cisplatin is a cytotoxic chemotherapic drug whose mechanism of action involves covalent binding to purine DNA bases, which primarily leads to cellular apoptosis. Despite significant advances in the development of novel cancer treatment, cisplatin is still used for tumors such as lung, head and neck, ovary, testicular, and bladder cancer, irrespective of its associated toxicity [142]. Among cisplatin-induced side effects, the most relevant are muscle mass loss and decline of muscle function. These muscle wasting conditions could significantly affect the patient’s quality of life, as well as therapy expected outcomes. Indeed, patients suffering from cisplatin-induced muscle wasting are often unable to complete treatment regimens and may require delays in treatment, dose limitation, or discontinuation of therapy [143]. It is well known that cisplatin administration promotes muscle wasting by activating a wide range of mechanisms. Indeed, muscle size and function are primarily affected by the activation of signaling pathways that have been implicated in promoting muscle atrophy and that are driven by processes that mainly impact on muscle protein homeostasis, calcium handling machinery, and mitochondrial metabolism. The dissection of the molecular pathways responsible for this condition gives the possibility to identify novel promising therapeutic targets for counteracting cisplatin-induced muscle dysfunction, such as ghrelin mimetics, D-methionine, taurine, and activins modulators. Although up to date there are no approved treatment for muscle wasting induced by cisplatin administration, all these proposed therapeutic strategies could hopefully pave the way to the identification of drugs effectively ameliorating the patients’ quality of life.

## Figures and Tables

**Table 1:** Ghrelin, GHS, D-methionine and Taurine: mechanisms of action in counteracting muscle-cisplatin induce toxicity.

Molecular Mechanisms of Cisplatin-Induced Muscle Toxicity	Specific Mechanism Prevented or Counteracted by Each Indicated Molecule			
	Ghrelin	GHS	D-Methionine	Taurine
Altered ubiquitin-proteasome system	√	√	√	
Altered autophagy				√
Inhibition of myogenic programme	√	√	√	√
Altered calcium homeostasis		√		
Mitochondrial damage		√		√
Oxidative stress		√		
Pro-inflammatory effect	√			
Altered lipid metabolism	√			

## Abbreviations

ACVR2B	activin receptor type 2B
aP2	adipocyte fatty acid binding protein
cisplatin	cis-diamine-dichloroplatinum (II)
CNS	central nervous system
CPT-1	carnitine palmitoyl transferase-1
DHPR	voltage-sensitive calcium channel
D-met	D-methionine
Drp1	dynamin-related protein 1
ECC	excitation–contraction coupling
ER	endoplasmic reticulum
FA	fatty acids
FAS	fatty acid synthase
FoxO3a	forkhead boxO
FSH	follicle-stimulating hormone
GDF11	growth differentiation factor-11
GHRP	growth hormone releasing peptides
GHS	growth hormone secretagogues
GHS-R1a	growth hormone secretagogues receptor 1 a
HSL	hormone sensitive lipase
IFN-γ	interferon-γ
IGF-1	insulin-like growth factor-1
IL-1	interleukin-1
IL-6	interleukin-6
LBM	lean body mass
LC3	lipidated microtubule-associated protein 1 light chain 3 alpha
MAFbx	muscle atrophy F-Box
MFN1	mitofusins 1
MFN2	mitofusins 2
MnSOD	mitochondrial superoxide dismutase.
Mstn	myostatin
mTOR	mammalian target of rapamycin
mPTP	mitochondrial permeability transition pore
MuRF-1	muscle ring-finger-1
NF-κB	prototypical nuclear factor kappa light-chain-enhancer of activated B cells
NRF-1	nuclear respiratory factor 1
Orai1	Ca^2+^ release-activated Ca^2+^ modulator 1
PGC-1α	peroxisome proliferator-activated receptor γ coactivator-1α
PI3K	phosphoinositide 3-kinase
PLC	phospholipase C
PRX III	peroxiredoxin III
ROS	reactive oxygen species
RYR	ryanodine receptor
SCD-1	stearoyl coenzyme A desaturase-1
SERCA	sarcoplasmic/endoplasmic reticulum Ca^2+^ATPase
Smad2/3	small mother against decapentaplegic
SOCE	store-operated calcium entry
SOD	superoxide dismutase
SR	sarcoplasmic reticulum
STAT	transcription factors of the signal transducer and activator of transcription
Stim1	stromal interaction molecule
TA	tibialis anterioris
Taurine	2-aminoethanesulfonic acid
TFAM	mitochondrial transcription factor A
TGF-β	transforming growth factor beta
TNF-α	tumor necrosis factor-α
UPP	ubiquitin-proteasome pathway
WAT	white adipose tissue

## References

[1] Kellad L. (2007). The resurgence of platinum-based cancer chemotherapy. Nat. Rev. Cancer.

[2] Frezza M., Hindo S., Chen D., Davenport A., Schmitt S., Tomco D., Dou Q.P. (2010). Novel metals and metal complexes as platform for cancer therapy. Curr. Pharm. Des..

[3] Acharyya S., Butchbach M.E., Sahenk Z., Wang H., Saji M., Carathers M., Ringel M.D., Skipworth R.J., Fearon K.C., Hollingsworth M.A. (2005). Dystrophin glycoprotein complex dysfunction: A regulatory link between muscular dystrophy and cancer cachexia. Cancer Cell.

[4] Berardi E. (2017). Muscular Dystrophies and Cancer Cachexia: Similarities in Chronic Skeletal Muscle Degeneration. J. Funct. Morphol. Kinesiol..

[5] Garcia J.M., Cata J.P., Dougherty P.M., Smith R.G. (2008). Ghrelin prevents cisplatin-induced mechanical hyperalgesia and cachexia. Endocrinology.

[6] Dickey D.T., Muldoon L.L., Doolittle N.D., Peterson D.R., Kraemer D.F., Neuwelt E.A. (2008). Effect of N-acetylcysteine route of administration on chemoprotection against cisplatin-induced toxicity in rat models. Cancer Chemother. Pharmacol..

[7] Sakai H., Sagara A., Arakawa K., Sugiyama R., Hirosaki A., Takase K., Jo A., Sato K., Chiba Y., Yamazaki M. (2014). Mechanisms of cisplatin-induced muscle atrophy. Toxicol. Appl. Pharmacol..

[8] Bresciani E., Rizzi L., Molteni L., Ravelli M., Liantonio A., Ben Haj Salah K., Fehrentz J.A., Martinez J., Omeljaniuk R.J., Biagini G. (2017). JMV2894, a novel growth hormone secretagogue, accelerates body mass recovery in an experimental model of cachexia. Endocrine.

[9] Conte E., Camerino G.M., Mele A., De Bellis M., Pierno S., Rana F., Fonzino A., Caloiero R., Rizzi L., Bresciani E. (2017). Growth hormone secretagogues prevent dysregulation of skeletal muscle calcium homeostasis in a rat model of cisplatin-induced cachexia. J. Cachexia Sarcopenia Muscle.

[10] Fearon K., Strasser F., Anker S.D., Bosaeus I., Bruera E., Fainsinger R.L., Jatoi A., Loprinzi C., MacDonald N., Mantovani G. (2011). Definition and classification of cancer cachexia: An international consensus. Lancet Oncol..

[11] Nicolini A., Ferrari P., Masoni M.C., Fini M., Pagani S., Giampietro O., Carpi A. (2013). Malnutrition, anorexia and cachexia in cancer patients: A mini-review on pathogenesis and treatment. Biomed. Pharmcother..

[12] Khalil R. (2018). Ubiquitin-Proteasome Pathway and Muscle Atrophy. Adv. Exp. Med. Biol..

[13] Bodine S.C., Baehr L.M. (2014). Skeletal muscle atrophy and the E3 ubiquitin ligases MuRF1 and MAFbx/atrogin-1. Am. J. Physiol. Endocrinol. Metab..

[14] Dehoux M., Van Beneden R., Pasko N., Lause P., Verniers J., Underwood L., Ketelslegers J.M., Thissen J.P. (2004). Role of the insulin-like growth factor I decline in the induction of atrogin-1/MAFbx during fasting and diabetes. Endocrinology.

[15] Clavel S., Coldefy A.S., Kurkdjian E., Salles J., Margaritis I., Derijard B. (2006). Atrophy-related ubiquitin ligases, atrogin-1 and MuRF1 are up-regulated in aged rat Tibialis Anterior muscle. Mech. Ageing Dev..

[16] Zhang Y., Gan B., Liu D., Paik J.H. (2011). FoxO family members in cancer. Cancer Biol..

[17] Penna F., Costamagna D., Fanzani A., Bonelli G., Baccino F.M., Costelli P. (2010). Muscle wasting and impaired myogenesis in tumor bearing mice are prevented by ERK inhibition. PLoS ONE.

[18] Layne M.D., Farmer S.R. (1999). Tumor necrosis factor-alpha and basic fibroblast growth factor differentially inhibit the insulin-like growth factor-I induced expression of myogenin in C2C12 myoblasts. Exp. Cell Res..

[19] Chen J.A., Splenser A., Guillory B., Luo J., Mendiratta M., Belinova B., Halder T., Zhang G., Li Y.P., Garcia J.M. (2015). Garcia Ghrelin prevents tumour- and cisplatin-induced muscle wasting: Characterization of multiple mechanisms involved. J. Cachexia Sarcopenia Muscle.

[20] Banerjee A., Guttridge D.C. (2012). Mechanisms for maintaining muscle. Curr. Opin. Support. Palliat. Care.

[21] Harish P., Malerba A., Lu-Nguyen N., Forrest L., Cappellari O., Roth F., Trollet C., Popplewell L., Dickson G. (2019). Inhibition of myostatin improves muscle atrophy in oculopharyngeal muscular dystrophy (OPMD). J. Cachexia Sarcopenia Muscle.

[22] Chen J.L., Walton K.L., Al-Musawi S.L., Kelly E.K., Qian H., La M., Lu L., Lovrecz G., Ziemann M., Lazarus R. (2015). Development of novel activin-targeted therapeutics. Mol. Ther..

[23] Trendelenburg A.U., Meyer A., Rohner D., Boyle J., Hatakeyama S., Glass D.J. (2009). Myostatin reduces Akt/TORC1/p70S6K signaling, inhibiting myoblast differentiation and myotube size. Am. J. Physiol. Cell Physiol..

[24] Porcelli L., Quatrale A.E., Mantuano P., Silvestris N., Rolland J.F., Biancolillo L., Paradiso A., Azzariti A. (2013). Synergistic antiproliferative and antiangiogenic effects of EGFR and mTOR inhibitors. Curr. Pharm. Des..

[25] Cai D., Frantz J.D., Tawa N.E., Melendez P.A., Oh B.C., Lidov H.G., Hasselgren P.O., Frontera W.R., Lee J., Glass D.J. (2004). IKKbeta/NFkappaB activation causes severe muscle wasting in mice. Cell.

[26] Schakman O., Dehoux M., Bouchuari S., Delaere S., Lause P., Decroly N., Shoelson S.E., Thissen J.P. (2012). Role of IGF-I and the TNFα/NF-kB pathway in the induction of muscle atrogenes by acute inflammation. Am. J. Physiol. Endocrinol. Metab..

[27] Li H., Malhotra S., Kumar A. (2008). Nuclear factor-kappa B signaling in skeletal muscle atrophy. J. Mol. Med..

[28] Camp E.R., Li J., Minnich D.J., Brank A., Moldawer L.L., MacKay S.L., Hochwald S.N. (2004). Inducible nuclear factor-kappaB activation contributes to chemotherapy resistance in gastric cancer. J. Am. Coll. Surg..

[29] Damrauer J.S., Stadler M.E., Acharyya S., Baldwin A.S. (2008). Chemotherapy-induced muscle wasting: Association with NF-kB and cancer cachexia. Basic Appl. Myol..

[30] Zhang Y., Pan X., Sun Y., Geng Y.J., Yu X.Y., Li Y. (2018). The Molecular Mechanisms and Prevention Principles of Muscle Atrophy in Aging. Adv. Exp. Med. Biol..

[31] Galluzzi L., Baehrecke E.H., Ballabio A., Boya P., Bravo-San Pedro J.M., Cecconi F., Choi A.M., Chu C.T., Codogno P., Colombo M.I. (2017). Molecular definitions of autophagy and related processes. EMBO J..

[32] Mammucari C., Milan G., Romanello V., Masiero E., Rudolf R., Del Piccolo P., Burden S.J., Di Lisi R., Sandri C., Zhao J. (2007). FoxO_3_ controls autophagy in skeletal muscle in vivo. Cell Metab..

[33] Sandri M., Sandri C., Gilbert A., Skurk C., Calabria E., Picard A., Walsh K., Schiaffino S., Lecker S.H., Goldberg A.L. (2004). Foxo transcription factors induce the atrophy-related ubiquitin ligase atrogin-1 and cause skeletal muscle atrophy. Cell.

[34] Brunet A., Bonni A., Zigmond M.J., Lin M.Z., Juo P., Hu L.S., Anderson M.J., Arden K.C., Blenis J., Greenberg M.E. (1999). Akt promotes cell survival by phosphory-lating and inhibiting a Forkhead transcription factor. Cell.

[35] Sandri M. (2010). Autophagy in Skeletal Muscle. FEBS Lett..

[36] Sirago G., Conte E., Fracasso F., Cormio A., Fehrentz J.A., Martinez J., Musicco C., Camerino G.M., Fonzino A., Rizzi L. (2017). Growth hormone secretagogues hexarelin and JMV2894 protect skeletal muscle from mitochondrial damages in a rat model of cisplatin-induced cachexia. Sci. Rep..

[37] Fanzani A., Zanola A., Rovetta F., Rossi S., Aleo M.F. (2011). Cisplatin triggers atrophy of skeletal C2C12 myotubes via impairment of Akt signalling pathway and subsequent increment activity of proteasome and autophagy systems. Toxicol. Appl. Pharmacol..

[38] Gehlert S., Bloch W., Suhr F. (2015). Ca^2+^-dependent regulations and signaling in skeletal muscle: From electro-mechanical coupling to adaptation. Int. J. Mol. Sci..

[39] Parekh A.B., Penner R. (1997). Store depletion and calcium influx. Physiol. Rev..

[40] Kurebayashi N., Ogawa Y. (2001). Depletion of Ca^2+^ in the sarcoplasmic reticulum stimulates Ca^2+^ entry into mouse skeletal muscle fibres. J. Physiol..

[41] Brunetti O., Imbrici P., Botti F.M., Pettorossi V.E., D’Adamo M.C., Valentino M., Zammit C., Mora M., Gibertini S., Di Giovanni G. (2012). Kv1.1 knock-in ataxic mice exhibit spontaneous myokymic activity exacerbated by fatigue, ischemia and low temperature. Neurobiol. Dis..

[42] Graves T.D., Imbrici P., Kors E.E., Terwindt G.M., Eunson L.H., Frants R.R., Haan J., Ferrari M.D., Goadsby P.J., Hanna M.G. (2008). Premature stop codons in a facilitating EF-hand splice variant of CaV2.1 cause episodic ataxia type 2. Neurobiol. Dis..

[43] Agrawal A., Rathor R., Suryakumar G. (2017). Oxidative protein modification alters proteostasis under acute hypobaric hypoxia in skeletal muscles: A comprehensive in vivo study. Cell Stress Chaperones.

[44] Agrawal A., Suryakumar G., Rathor R. (2018). Role of defective Ca^2+^ signaling in skeletal muscle weakness: Pharmacological implications. J. Cell. Commun. Signal..

[45] Berchtold M.W., Brinkmeier H., Müntener M. (2000). Calcium ion in skeletal muscle: Its crucial role for muscle function, plasticity, and disease. Physiol. Rev..

[46] Sorimachi H., Ono Y. (2012). Regulation and physiological roles of the calpain system in muscular disorders. Cardiovasc. Res..

[47] Romanello V., Sandri M. (2016). Mitochondrial quality control and muscle mass maintenance. Front. Physiol..

[48] Carter H.N., Chen C.C., Hood D.A. (2015). Mitochondria, muscle health, and exercise with advancing age. Physiology.

[49] Youle R.J., van der Bliek A.M. (2012). Mitochondrial fission, fusion, and stress. Science.

[50] Che R., Yuan Y., Huang S., Zhang A. (2014). Mitochondrial dysfunction in the pathophysiology of renal diseases. Am. J. Physiol. Ren. Physiol..

[51] Nan J., Zhu W., Rahman M.S., Liu M., Li D., Su S., Zhang N., Hu X., Yu H., Gupta M.P. (2017). Molecular regulation of mitochondrial dynamics in cardiac disease. Biochim. Biophys. Acta.

[52] Powers S.K., Wiggs M.P., Duarte J.A., Zergeroglu A.M., Demirel H.A. (2012). Mitochondrial signaling contributes to disuse muscle atrophy. Am. J. Physiol. Endocrinol. Metab..

[53] Wood Z.A., Poole L.B., Karplus P.A. (2003). Peroxiredoxin evolution and the regulation of hydrogen peroxide signaling. Science.

[54] Azadmanesh J., Borgstahl G. (2018). A review of the catalytic mechanism of human manganese superoxide dismutase. Antioxidants.

[55] Zhang L., Du J., Hu Z., Han G., Delafontaine P., Garcia G., Mitch W.E. (2009). IL-6 and serum amyloid A synergy mediates angiotensin II-induced muscle wasting. J. Am. Soc. Nephrol..

[56] Moreira-Pais A., Ferreira R., da Costa R.G. (2018). Platinum-induced Muscle Wasting in Cancer Chemotherapy: Mechanisms and Potential Targets for Therapeutic Intervention. Life Sci..

[57] Legaspi A., Jeevanandam M., Starnes H.F., Brennan M.F. (1987). Whole body lipid and energy metabolism in the cancer patient. Metabolism.

[58] Klein S., Wolfe R.R. (1990). Whole-body lipolysis and triglyceride-fatty acid cycling in cachectic patients with esophageal cancer. J. Clin. Investig..

[59] Jeevanandam M., Horowitz G.D., Lowry S.F., Brennan M.F. (1986). Cancer cachexia and the rate of whole body lipolysis in man. Metabolism.

[60] Ryden M., Agustsson T., Laurencikiene J., Britton T., Sjolin E., Isaksson B., Permert J., Arner P. (2008). Lipolysis not inflammation, cell death, or lipogenesis is involved in adipose tissue loss in cancer cachexia. Cancer.

[61] Das S.K., Eder S., Schauer S., Diwoky C., Temmel H., Guertl B., Gorkiewicz G., Tamilarasan K.P., Kumari P., Trauner M. (2011). Adipose triglyceride lipase contributes to cancer-associated cachexia. Science.

[62] Garcia J.M., Scherer T., Chen J.A., Guillory B., Nassif A., Papusha V., Smiechowska J., Asnicar M., Buettner C., Smith R.G. (2013). Inhibition of cisplatin-induced lipid catabolism and weight loss by ghrelin in male mice. Endocrinology.

[63] Hertzel A.V., Hellberg K., Reynolds J.M., Kruse A.C., Juhlmann B.E., Smith A.J., Sanders M.A., Ohlendorf D.H., Suttles J., Bernlohr D.A. (2009). Identification and characterization of a small molecule inhibitor of fatty acid binding proteins. J. Med. Chem..

[64] Makowski L., Boord J.B., Maeda K., Babaev V.R., Uysal K.T., Morgan M.A., Parker R.A., Suttles J., Fazio S., Hotamisligil G.S. (2001). Lack of macrophage fatty-acid-binding protein aP2 protects mice deficient in apolipoprotein E against atherosclerosis. Nat. Med..

[65] Shen W.J., Sridhar K., Bernlohr D.A., Kraemer F.B. (1999). Interaction of rat hormone-sensitive lipase with adipocyte lipid-binding protein. Proc. Natl. Acad. Sci. USA.

[66] Cooper C., Burden S.T., Cheng H., Molassiotis A. (2015). Understanding and managing cancer-related weight loss and anorexia: Insights from a systematic review of qualitative research. J. Cachexia Sarcopenia Muscle.

[67] Serrenho D., Santos S.D., Carvalho A.L. (2019). The role of gherlin in regulating synaptic function and plasticity of feeding associated circuits. Front. Cell. Neurosci..

[68] Lv Y., Liang T., Wang G., Li Z. (2018). Ghrelin, a gastrointestinal hormone, regulates energy balance and lipid metabolism. Biosci. Rep..

[69] Davis J. (2018). Hunger, ghrelin and the gut. Brain Res..

[70] Porporato P.E., Filigheddu N., Reano S., Ferrara M., Angelino E., Gnocchi V.F., Prodam F., Ronchi G., Fagoonee S., Fornaro M. (2013). Acylated and unacylated ghrelin impair skeletal muscle atrophy in mice. J. Clin. Investig..

[71] Sugiyama M., Yamaki A., Furuya M., Inomata N., Minamitake Y., Ohsuye K., Kangawa K. (2012). Ghrelin improves body weight loss and skeletal muscle catabolism associated with angiotensin II-induced cachexia in mice. Regul. Pept..

[72] Hiura Y., Takiguchi S., Yamamoto K., Takahashi T., Kurokawa Y., Yamasaki M., Nakajima K., Miyata H., Fujiwara Y., Mori M. (2012). Effects of ghrelin administration during chemotherapy with advanced esophageal cancer patients: A prospective, randomized, placebo-controlled phase 2 study. Cancer.

[73] Bowers C.Y. (1998). Growth hormone-releasing peptide (GHRP). Cell. Mol. Life Sci..

[74] Smith R.G., van der Ploeg L.H., Howard A.D., Feighner S.D., Cheng K., Hickey G.J., Wyvratt M.J., Fisher M.H., Nargund R.P., Patchett A.A. (1997). Peptidomimetic regulation of growth hormonesecretion. Endocr. Rev..

[75] Ghigo E., Arvat E., Giordano R., Broglio F., Gianotti L., Maccario M., Bisi G., Graziani A., Papotti M., Muccioli G. (2001). Biologic activities of growth hormone secretagogues in humans. Endocrine.

[76] Rivier J., Spiess J., Thorner M.O., Vale W. (1982). Characterization of a growth hormone-releasing factor from a human pancreatic islet tumor. Nature.

[77] Kojima M., Hosoda H., Date Y., Nakazato M., Matsuo H., Kangawa K. (1999). Ghrelin is a growth-hormone-releasing acylated peptide from stomach. Nature.

[78] Arvat E., Broglio F., Aimaretti G., Benso A., Giordano R., Deghenghi R., Ghigo E. (2002). Ghrelin and synthetic GH secretagogues. Best Pract. Res. Clin. Endocrinol. Metab..

[79] Müller D., Nogueiras R., Andermann M.L., Andrews Z.B., Anker S.D., Argente J., Batterham R.L., Benoit S.C., Bowers C.Y., Broglio F. (2015). Ghrelin. Mol. Metab..

[80] Howard A.D., Feighner S.D., Cully D.F., Arena J.P., Liberator P.A., Rosenblum C.I., Hamelin M., Hreniuk D.L., Palyha O.C., Anderson J. (1996). A receptor in pituitary and hypothalamus that functions in growth hormone release. Science.

[81] Torsello A., Luoni M., Schweiger F., Grilli R., Guidi M., Bresciani E., Deghenghi R., Müller E.E., Locatelli V. (1998). Novel hexarelin analogs stimulate feeding in the rat through a mechanism not involving growth hormone release. Eur. J. Pharmacol..

[82] Bodart V., Febbraio M., Demers A., McNicoll N., Pohankova P., Perreault A., Sejlitz T., Escher E., Silverstein R.L., Lamontagne D. (2002). CD36 mediates the cardiovascular action of growth hormone-releasing peptides in the heart. Circ. Res..

[83] Rodrigue-Way A.A., Demers H., Ong A., Tremblay A. (2007). Growth hormone-releasing peptide promotes mitochondrial biogenesis and a fat burning-like phenotype through scavenger receptor CD36 in white adipocytes. Endocrinology.

[84] Muccioli G., Baragli A., Granata R., Papotti M., Ghigo E. (2007). Heterogeneity of ghrelin/growth hormone secretagogue receptors. Toward the understanding of the molecular identity of novel ghrelin/GHS receptors. Neuroendocrinology.

[85] Granado M., Priego T., Martín A.I., Villanúa M.A., López-Calderón A. (2005). Anti-inflammatory effect of the ghrelin agonist growth hormone-releasing peptide-2 (GHRP-2) in arthritic rats. Am. J. Physiol. Endocrinol. Metab..

[86] Granado M., Martín A.I., López-Menduiña M., López-Calderón A., Villanúa M.A. (2008). GH-releasing peptide-2 administration prevents liver inflammatory response in endotoxemia. Am. J. Physiol. Endocrinol. Metab..

[87] Bulgarelli I., Tamiazzo L., Bresciani E., Rapetti D., Caporali S., Lattuada D., Locatelli V., Torsello A. (2009). Desacyl-ghrelin and synthetic GH-secretagogues modulate the production of inflammatory cytokines in mouse microglia cells stimulated by beta-amyloid fibrils. J. Neurosci. Res..

[88] Lucchi C., Curia G., Vinet J., Gualtieri F., Bresciani E., Locatelli V., Torsello A., Biagini G. (2013). Protective but not anticonvulsant effects of ghrelin and JMV-1843 in the pilocarpine model of Status epilepticus. PLoS ONE.

[89] Moulin A., Demange L., Bergé G., Gagne D., Ryan J., Mousseaux D., Heitz A., Perrissoud D., Locatelli V., Torsello A. (2007). Toward potent ghrelin receptor ligands based on trisubstituted 1,2,4-triazole structure. 2. Synthesis and pharmacological in vitro and in vivo evaluations. J. Med. Chem..

[90] Cocchi D., Maccarinelli G., Sibilia V., Tulipano G., Torsello A., Pazzaglia U.E., Giustina A., Netti C. (2005). GH-releasing peptides and bone. J. Endocrinol. Investig..

[91] Sibilia V., Pagani F., Guidobono F., Locatelli V., Torsello A., Deghenghi R., Netti C. (2002). Evidence for a central inhibitory role of growth hormone secretagogues and ghrelin on gastric acid secretion in conscious rats. Neuroendocrinology.

[92] Sibilia V., Torsello A., Pagani F., Rapetti D., Lattuada N., Locatelli V., Bulgarelli I., Guidobono F., Netti C. (2004). Effects of hexarelin against acid-independent and acid-dependent ulcerogens in the rat. Peptides.

[93] Karasawa H., Pietra C., Giuliano C., Garcia-Rubio S., Xu X., Yakabi S., Taché Y., Wang L. (2014). New ghrelin agonist, HM01 alleviates constipation and L-dopa-delayed gastric emptying in 6-hydroxydopamine rat model of Parkinson’s disease. Neurogastroenterol. Motil..

[94] Xu X.B., Pang J.J., Cao J.M., Ni C., Xu R.K., Peng X.Z., Yu X.X., Guo S., Chen M.C., Chen C. (2005). GH-releasing peptides improve cardiac dysfunction and cachexia and suppress stress-related hormones and cardiomyocyte apoptosis in rats with heart failure. Am. J. Physiol. Heart Circ. Physiol..

[95] Ma Y., Zhang L., Launikonis B.S., Chen C. (2012). Growth hormone secretagogues preserve the electrophysiological properties of mouse cardiomyocytes isolated from in vitro ischemia/reperfusion heart. Endocrinology.

[96] Torsello A., Rossoni G., Locatelli V., De Gennaro Colonna V., Bernareggi M., Francolini M., Müller E.E., Berti F. (2001). Hexarelin, but not growth hormone, protects heart from damage induced in vitro by calcium deprivation replenishment. Endocrine.

[97] Torsello A., Bresciani E., Rossoni G., Avallone R., Tulipano G., Cocchi D., Bulgarelli I., Deghenghi R., Berti F., Locatelli V. (2003). Ghrelin plays a minor role in the physiological control of cardiac function in the rat. Endocrinology.

[98] McDonald H., Peart J., Kurniawan N., Galloway G., Royce S., Samuel C., Chen C. (2018). Hexarelin treatment preserves myocardial function and reduces cardiac fibrosis in a mouse model of acute myocardial infarction. Physiol. Rep..

[99] Broglio F., Guarracino F., Benso A., Gottero C., Prodam F., Granata R., Avogadri E., Muccioli G., Deghenghi R., Ghigo E. (2002). Effects of acute hexarelin administration on cardiac performance in patients with coronary artery disease during by-pass surgery. Eur. J. Pharmacol..

[100] Imazio M., Bobbio M., Broglio F., Benso A., Podio V., Valetto M.R., Bisi G., Ghigo E., Trevi G.P. (2002). GH-independent cardiotropic activities of hexarelin in patients with severe left ventricular dysfunction due to dilated and ischemic cardiomyopathy. Eur. J. Heart Fail..

[101] Borner T., Loi L., Pietra C., Giuliano C., Lutz T.A., Riediger T. (2016). The ghrelin receptor agonist HM01 mimics the neuronal effects of ghrelin in the arcuate nucleus and attenuates anorexia-cachexia syndrome in tumor-bearing rats. Am. J. Physiol. Regul. Integr. Comp. Physiol..

[102] Villars F.O., Pietra C., Giuliano C., Lutz T.A., Riediger T. (2017). Oral Treatment with the Ghrelin Receptor Agonist HM01 Attenuates Cachexia in Mice Bearing Colon-26 (C26) Tumors. Int. J. Mol. Sci..

[103] Yoshimura M., Shiomi Y., Ohira Y., Takei M., Tanaka T. (2017). Z-505 hydrochloride, an orally active ghrelin agonist, attenuates the progression of cancer cachexia via anabolic hormones in Colon 26 tumor-bearing mice. Eur. J. Pharmacol..

[104] Deghenghi R., Cananzi M.M., Torsello A., Battisti C., Müller E.E., Locatelli V. (1994). GH-releasing activity of hexarelin, a new growth hormone releasing peptide, in infant and adult rats. Life Sci..

[105] Rom O., Reznick A.Z. (2016). The role of E3 ubiquitin-ligases MuRF-1 and MAFbx in loss of skeletal muscle mass. Free Radic. Biol. Med..

[106] Ji L.L., Kang C. (2015). Role of PGC-1α in sarcopenia: Etiology and potential intervention—A mini-review. Gerontology.

[107] Nishie K., Yamamoto S., Nagata C., Koizumi T., Hanaoka M. (2017). Anamorelin for advanced non-small-cell lung cancer with cachexia: Systematic review and meta-analysis. Lung Cancer.

[108] Baracos V.E., Mazurak V.C., Bhullar A.S. (2019). Cancer cachexia is defined by an ongoing loss of skeletal muscle mass. Ann. Palliat. Med..

[109] Sacheck J.M., Ohtsuka A., McLary S.C., Goldberg A.L., Sever S., White D. (2004). IGF-I stimulates muscle growth by suppressing protein breakdown and expression of atrophy-related ubiquitin ligases, atrogin-1 and MuRF1. Am. J. Physiol. Endocrinol. Metab..

[110] Metayer S., Seiliez I., Collin A., Duchêne S., Mercier Y., Geraert P.A., Tesseraud S. (2008). Mechanisms through which sulfur amino acids control protein metabolism and oxidative status. J. Nutr. Biochem..

[111] Willke T. (2014). Methionine production—A critical review. Appl. Microbiol. Biotechnol..

[112] Gonçalves M., Silveira A., Teixeira A., Hyppolito M. (2013). Mechanisms of cisplatin ototoxicity: Theoretical review. J. Laryngol. Otol..

[113] Lo W.C., Chang C.M., Liao L.J., Wang C.T., Young Y.H., Chang Y.L., Cheng P.W. (2015). Assessment of d-methionine protecting cisplatin-induced otolith toxicity by vestibular-evoked myogenic potential tests, ATPase activities and oxidative state in guinea pigs. Neurotoxicol. Teratol..

[114] Campbell K.C., Meech R.P., Rybak L.P., Hughes L.F. (2003). The effect of D-methionine on cochlear oxidative state with and without cisplatin administration: Mechanisms of otoprotection. J. Am. Acad. Audiol..

[115] Cheng P.W., Liu S.H., Young Y.H., Lin-Shiau S.Y. (2006). D-Methionine attenuated cisplatin-induced vestibulotoxicity through altering ATPase activities and oxidative stress in guinea pigs. Toxicol. Appl. Pharmacol..

[116] Hamstra D.A., Lee K.C., Eisbruch A., Sunkara P., Borgonha S., Phillip B., Campbell K.C.M., Ross B.D., Rehemtulla A. (2018). Double-blind placebo-controlled multicenter phase II trial to evaluate d-methionine in preventing/reducing oral mucositis induced by radiation and chemotherapy for head and neck cancer. Head Neck.

[117] Liao Y., Lu X., Lu C., Li G., Jin Y., Tang H. (2008). Selection of agents for prevention of cisplatin-induced hepatotoxicity. Pharmacol. Res..

[118] Hinduja S., Kraus K.S., Manohar S., Salvi R.J. (2015). D-methionine protects against cisplatin-induced neurotoxicity in the hippocampus of the adult rat. Neurotox. Res..

[119] Lin M.T., Ko J.L., Liu T.C., Chao P.T., Ou C.C. (2018). Protective Effect of d-Methionine on Body Weight Loss, Anorexia, and Nephrotoxicity in Cisplatin-Induced Chronic Toxicity in Rats. Integr. Cancer.

[120] Wu C.H., Ko J.L., Liao J.M., Huang S.S., Lin M.Y., Lee L.H., Chang L.Y., Ou C.C. (2019). d-methionine alleviates cisplatin-induced mucositis by restoring the gut microbiota structure and improving intestinal inflammation. Adv. Med. Oncol..

[121] Wu C.T., Liao J.M., Ko J.L., Lee Y.L., Chang H.Y., Wu C.H., Ou C.C. (2019). D-Methionine Ameliorates Cisplatin-Induced Muscle Atrophy via Inhibition of Muscle Degradation Pathway. Integr. Cancer.

[122] Wo’jcik O.P., Koenig K.L., Zeleniuch-Jacquotte A., Costa M., Chen Y. (2010). The potential protective effects of taurine on coronary heart disease. Atherosclerosis.

[123] Rovetta F., Stacchiotti A., Consiglio A., Cadei M., Grigolato P.G., Lavazza A., Rezzani R., Aleo M.F. (2012). ER signaling regulation drives the switch between autophagy and apoptosis in NRK-52E cells exposed to cisplatin. Exp. Cell Res..

[124] De Luca A., Pierno S., Tricarico D., Desaphy J.-F., Liantonio A., Barbieri M., Camerino C., Montanari L., Conte Camerino D. (2000). Taurine and skeletal muscle ion channels. Adv. Exp. Med. Biol..

[125] De Luca A., Pierno S., Conte Camerino D. (2015). Taurine: The appeal of a safe amino acid for skeletal muscle disorders journal of trasnational medicine. J. Transl. Med..

[126] Goodman C.A., Hayes A., McKenna M.J. (2009). Dissociation between force and maximal Na^+^, K^+^—ATPase activity in rat fast-twitch skeletal muscle with fatiguing in vitro stimulation. Eur. J. Appl. Physiol..

[127] Stacchiotti A., Rovetta F., Ferroni M., Corsetti G., Lavazza A., Sberveglieri G., Aleo M.F. (2014). Taurine rescues cisplatin-induced muscle atrophy in vitro: A morphological study. Oxidative Med. Cell. Longev..

[128] Lee S.J., McPherron A.C. (2001). Regulation of myostatin activity and muscle growth. Proc. Natl. Acad. Sci. USA.

[129] McPherron A.C., Lawler A.M., Lee S.J. (1997). Regulation of skeletal muscle mass in mice by a new TGF-beta superfamily members. Nature.

[130] Vale W., Rivier J., Vaughan J., McClintock R., Corrigan A., Woo W., Karr D., Spiess J. (1986). Purification and characterization of an FSH releasing protein from porcine ovarian follicular fluid. Nature.

[131] Ling N., Ying S.Y., Ueno N., Shimasaki S., Esch F., Hotta M., Guillemin R. (1986). A homodimer of the beta-subunits of inhibin A stimulates the secretion of pituitary follicle stimulating hormone. Biochem. Biophys. Res. Commun..

[132] Namwanje M., Brown C.W. (2016). Activins and Inhibins: Roles in Development, Physiology, and Disease. Cold Spring Harb. Perspect. Biol..

[133] Souza T.A., Chen X., Guo Y., Sava P., Zhang J., Hill J.J., Yaworsky P.J., Qiu Y. (2008). Proteomic identification and functional validation of activins and bone morphogenetic protein 11 as candidate novel muscle mass regulators. Mol. Endocrinol..

[134] Lee S.J., Reed L.A., Davies M.V., Girgenrath S., Goad M.E., Tomkinson K.N., Wright J.F., Barker C., Ehrmantraut G., Holmstrom J. (2005). Regulation of muscle growth by multiple ligands signaling through activin type II receptors. Proc. Natl. Acad. Sci. USA.

[135] Tan C.K., Leuenberger N., Tan M.J., Yan Y.W., Chen Y., Kambadur R., Wahli W., Tan N.S. (2011). Smad3 deficiency in mice protects against insulin resistance and obesity induced by a high-fat diet. Diabetes.

[136] Bogdanovich S., Krag T.O., Barton E.R., Morris L.D., Whittemore L.A., Ahima R.S., Khurana T.S. (2002). Functional improvement of dystrophic muscle by myostatin blockade. Nature.

[137] Zhou X., Wang J.L., Lu J., Song Y., Kwak K.S., Jiao Q., Rosenfeld R., Chen Q., Boone T., Simonet W.S. (2010). Reversal of cancer cachexia and muscle wasting by ActRIIB antagonism leads to prolonged survival. Cell.

[138] Relizani K., Mouisel E., Giannesini B., Hourdé C., Patel K., Morales Gonzalez S., Jülich K., Vignaud A., Piétri-Rouxel F., Fortin D. (2014). Blockade of ActRIIB signaling triggers muscle fatigability and metabolic myopathy. Mol. Ther..

[139] Latres E., Pangilinan J., Miloscio L., Bauerlein R., Na E., Potocky T.B., Huang Y., Eckersdorff M., Rafique A., Mastaitis J. (2015). Myostatin blockade with a fully human monoclonal antibody induces muscle hypertrophy and reverses muscle atrophy in young and aged mice. Skelet. Muscle.

[140] Becker C., Lord S.R., Studenski S.A., Warden S.J., Fielding R.A., Recknor C.P., Hochberg M.C., Ferrari S.L., Blain H., Binder E.F. (2015). Myostatin antibody (LY2495655) in older weak fallers: A proof-of-concept, randomised, phase 2 trial. Lancet Diabetes Endocrinol..

[141] Hatakeyama S., Summermatter S., Jourdain M., Melly S., Minetti G.C., Lach-Trifilieff E. (2016). ActRII blockade protects mice from cancer cachexia and prolongs survival in the presence of anti-cancer treatments. Skelet. Muscle.

[142] Argiles J.M., Busquets S., Stemmler B., Lopez-Soriano F.J. (2014). Cancer cachexia: Understanding the molecular basis. Nat. Rev. Cancer.

[143] Cleeland C.S., Allen J.D., Roberts S.A. (2012). Reducing the toxicity of cancer therapy: Recognizing needs, taking action. Nat. Rev. Clin. Oncol..

